# Structural and Functional Plasticity in the Regenerating Olfactory System of the Migratory Locust

**DOI:** 10.3389/fphys.2020.608661

**Published:** 2020-12-03

**Authors:** Gerd Bicker, Michael Stern

**Affiliations:** Division of Cell Biology, Institute of Physiology and Cell Biology, University of Veterinary Medicine Hannover, Hannover, Germany

**Keywords:** antennal lobe, mushroom body, fasciclin I, semaphorin 1a, field potential oscillations

## Abstract

Regeneration after injury is accompanied by transient and lasting changes in the neuroarchitecture of the nervous system and, thus, a form of structural plasticity. In this review, we introduce the olfactory pathway of a particular insect as a convenient model to visualize neural regeneration at an anatomical level and study functional recovery at an electrophysiological level. The olfactory pathway of the locust (*Locusta migratoria*) is characterized by a multiglomerular innervation of the antennal lobe by olfactory receptor neurons. These olfactory afferents were axotomized by crushing the base of the antenna. The resulting degeneration and regeneration in the antennal lobe could be quantified by size measurements, dye labeling, and immunofluorescence staining of cell surface proteins implicated in axonal guidance during development. Within 3 days post lesion, the antennal lobe volume was reduced by 30% and from then onward regained size back to normal by 2 weeks post injury. The majority of regenerating olfactory receptor axons reinnervated the glomeruli of the antennal lobe. A few regenerating axons project erroneously into the mushroom body on a pathway that is normally chosen by second-order projection neurons. Based on intracellular responses of antennal lobe output neurons to odor stimulation, regenerated fibers establish functional synapses again. Following complete absence after nerve crush, responses to odor stimuli return to control level within 10–14 days. On average, regeneration of afferents, and re-established synaptic connections appear faster in younger fifth instar nymphs than in adults. The initial degeneration of olfactory receptor axons has a trans-synaptic effect on a second order brain center, leading to a transient size reduction of the mushroom body calyx. Odor-evoked oscillating field potentials, absent after nerve crush, were restored in the calyx, indicative of regenerative processes in the network architecture. We conclude that axonal regeneration in the locust olfactory system appears to be possible, precise, and fast, opening an avenue for future mechanistic studies. As a perspective of biomedical importance, the current evidence for nitric oxide/cGMP signaling as positive regulator of axon regeneration in connectives of the ventral nerve cord is considered in light of particular regeneration studies in vertebrate central nervous systems.

## Introduction

Central nervous systems are capable to reorganize their anatomical connectivity as a consequence of disruption or manipulation of sensory input. Insects are excellent models for studying such structural plasticity of the nervous system. Insect brains retain plastic capacities during adulthood, such as changes in neuropil volume and alterations in synaptic circuitry (Groh and Meinertzhagen, 2010; Kremer et al., 2010; Sugie et al., 2018). These characteristics support age-dependent, experience-dependent, and behavioral-coupled reorganization of the nervous system (Withers et al., 1993; Heisenberg et al., 1995; Gronenberg et al., 1996; Cayre et al., 2005; Ott and Rogers, 2010; Eriksson et al., 2019). A biomedical aspect relates to the plastic changes occurring in the nervous system after injury. Whereas the regenerative capacity of the central nervous system (CNS) in higher vertebrates is rather low (Ramon y Cajal, 1928; Rossignol et al., 2007), some insects are quite capable of neuronal regeneration both in the peripheral and the CNS (Spira et al., 1987; Stern et al., 1997; Jacobs and Lakes-Harlan, 1999; Pätschke et al., 2004; Ayaz et al., 2008; Stern and Bicker, 2008). Thus, core requirements for regeneration can be analyzed in insect nervous systems without the need to take myelin-associated inhibitory factors such as NOGO-A or MAG (Schwab and Strittmatter, 2014) into consideration, that are characteristics of the mature vertebrate neural tissue.

In the last decade, research into the mechanistics of peripheral nervous system (PNS) regeneration has made some remarkable progress in the genetic model insect, Drosophila melanogaster (reviewed by Brace and DiAntonio, 2017). Since the D. melanogaster larva is translucent, peripheral nerve injury can be delivered by pinching the cuticle and nerve with a sharp pair of forceps, severing both sensory and motor axons. Several studies deal with the ensuing degeneration of the separated distal segment, and molecules interfering with such degeneration such as the ubiquitin ligase, highwire (Xiong et al., 2012) or the NAD^+^ synthesizing axonal survival factor NMNAT2 (Walker et al., 2017). Some of these factors, e.g., the dual leucine zipper-containing kinase (DLK, Wallenda in D. melanogaster), interact both with distal segment degeneration (Brace et al., 2014) and initiation of regenerative mechanisms, like sprouting, in the proximal segment when activated by experimental elimination of the spectraplakin short stop (Valakh et al., 2013). Nevertheless, motor axons do not successfully navigate across the injury site using the crush paradigm in the D. melanogaster larva (Xiong et al., 2012). A second larval regeneration model in Drosophila melanogaster employs laser transection of dendrites and axons of genetically GFP-labeled sensory dendritic arborization (da) neurons (Song et al., 2012). Using this injury paradigm, the involvement of several cell intrinsic signaling pathways in enhancing regenerative responses to axon injury have been demonstrated: the Pten/Akt pathway (Song et al., 2012), the RNA processing enzyme dRtca (Song et al., 2015), or the CamKII/NOS/PKG pathway activated via the mechanosensitive Ca^2+^ channel piezo (Song et al., 2019). In summary, interference with those neuron-intrinsic mechanisms led to moderate improvements of incomplete axonal regeneration into the CNS. However, genetic reprogramming of glial metabolism to increased glycolysis could promote both significant da neuron regeneration after laser injury in the ventral nerve cord and restoration of the specific avoidance behavior mediated by these neurons (Li et al., 2020). Less has, so far, been achieved regarding regeneration in the adult Drosophila melanogaster. In the PNS, regeneration of wing vein sensory axons can be studied after laser axotomy. Surprisingly, regeneration is inhibited in this paradigm by the c-Jun N-terminal kinase (JNK) pathway (Soares et al., 2014), which is usually necessary for axon extension during nervous system development in D. melanogaster and elsewhere (Xiong et al., 2012). There is also an example for regeneration in the adult D. melanogaster CNS. In explanted adult Drosophila melanogaster brains, Ayaz et al. (2008) have studied regeneration of a group of identified neurons after microsurgical axotomy. The limited regenerative capacity of these axons was promoted by constitutive activation of protein kinase A, and further enhanced by activation of the JNK pathway in this system. Taken together, promising approaches to elucidate mechanisms of regeneration processes are in progress, but a powerful model for functional regeneration in the adult D. melanogaster brain has yet to be developed.

## The Insect Olfactory System

Mature insect olfactory neuropils exhibit a remarkably high degree of structural plasticity associated with specific behaviors (Withers et al., 1993; Heisenberg et al., 1995; Ott and Rogers, 2010; Kraft et al., 2019).

**FIGURE 1:**
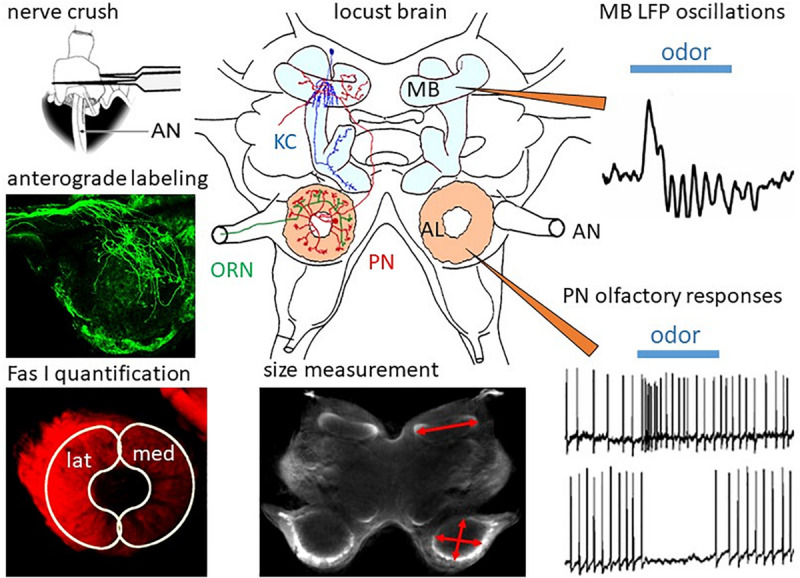
Regeneration in the olfactory system of the locust. In the locust brain, olfactory information from the ORNs projecting through the antennal nerves (AN) is processed in the ALs by local interneurons and PNs, which convey information to the mushroom bodies (MB) containing ca. 50,000 Kenyon cells (KC). After ORN axotomy by crushing the AN, degeneration and subsequent regeneration can be studied by measurement of MB and AL size, by anterograde labeling of ORNs, and by quantification of ORN projections into the lateral (lat) and medial (med) part of the AL by measuring immunofluorescence of their cell surface molecule, Fas I. Functional regeneration can be measured by intracellular recording odor-evoked responses from PNs and by recording of extracellular field potentials in the calyx of the MB. Adapted from Wasser et al. (2017) and Wasser and Stern (2017).

The locust is an accessible animal for the analysis of circuit mechanisms of olfactory coding strategies. Each locust antenna is equipped with 50,000 olfactory receptor neurons (ORNs) whose axons project into the antennal lobe (AL), the primary olfactory center (Ernst et al., 1977; Figure 1). The wiring of the insect AL shares common features with the vertebrate olfactory bulb (Strausfeld and Hildebrand, 1999). As in the olfactory bulb, in the insect AL ORNs synapse with local interneurons and olfactory projection neurons (vertebrates: mitral- and tufted cells) in spherical neuropil compartments termed glomeruli. The axons of the olfactory projection neurons (PNs) exit from the AL and project toward the mushroom body and the lateral horn (Laurent and Naraghi, 1994; Anton and Hansson, 1996; Hansson and Stensmyr, 2011). In the calycal compartment, PNs provide excitatory input to subsets of ca. 50,000 densely packed Kenyon cells, the intrinsic neurons of the mushroom body. Similar to mammals, in most insects each olfactory receptor neuron expresses only a single olfactory receptor gene. The receptor neurons expressing the same olfactory receptor gene converge their axons to the same glomerulus in the AL. Consequently, the number of receptor genes roughly corresponds to the number of glomeruli.

The locust uses a different wiring strategy in its olfactory system. Although the locust genome contains about 174 olfactory receptor genes (142 odorant receptors plus 32 ionotropic receptors, Wang et al., 2015), the AL comprises roughly 1,000 glomeruli (Ernst et al., 1977; Anton and Hansson, 1996). Each ORN axon branches in the AL, innervating multiple glomeruli (Ernst et al., 1977; Anton and Hansson, 1996; Hansson and Stensmyr, 2011). Each of the 830 PNs (Leitch and Laurent, 1996) receives its input from multiple glomeruli and electrophysiological estimates show that each Kenyon cell is contacted by half of the population of PNs (Jortner et al., 2007). This connectivity scheme of the locust olfactory system generates very selective responses by coincidence detection in the Kenyon cells, leading to sparse odor representations in the mushroom body (Jortner et al., 2007).

Since both the antennal sensory neurons and the PNs show a remarkable degree of convergence and also divergence to their postsynaptic partner neurons, one can assume that this type of connectivity pattern is not too rigidly specified. Indeed, in *Drosophila melanogaster* the synaptic connectivity from PNs to the Kenyon cells appears to be probabilistic (Murthy et al., 2008; Caron et al., 2013). At each larval molt during the hemimetabolous development of the locust, additional segments (annuli) are added to the flagellum of the antenna (Chapman, 2002; Boyan and Ehrhardt, 2020) which, in combination with growth of individual annuli, leads to an increase in the number of sensory neurons. The increasing number of ORN axons has to be incorporated into the already functioning circuitry. This requires some flexible synaptic reorganization, as observed, for instance, in the insect terminal ganglion, where a growing number of wind sensitive afferent connects to a fixed set of giant interneurons during postembryonic development (Chiba et al., 1988; Thompson et al., 1992). In the locust, the postembryonic remodeling of synaptic connectivity of prosternal filiform hair receptors has also been found (Pflüger et al., 1994). It is possible that a similar type of flexibility in the neuroachitecture of the AL, together with a somewhat loosely specified synaptic connectivity of the PNs to the mushroom body might be a helpful prerequisite for regeneration studies in the olfactory system.

## Regeneration in the Locust Olfactory System

In the following paragraphs, we will summarize some of the work in our own lab on regeneration in the olfactory system of adult and last larval instar locusts, which has been published in a series of papers (Eickhoff et al., 2012; Stern et al., 2012; Wasser et al., 2017; Wasser and Stern, 2017). The methodology is summarized in Figure 1.

In the locust antenna, ORN axons can be easily axotomized by crushing the scapus at the base of the antenna (Figure 1). The resulting changes in the brain can be quantified by size measurements on sections or, by using scanning laser optical tomography (SLOT, Lorbeer et al., 2011; Bode et al., 2020) in whole brains. The progression of degeneration and regeneration of ORN axons is traced by anterograde dye labeling through the antennal nerve and by quantitative immunofluorescence of the cell surface marker, Fasciclin 1 (Fas I). Functional regeneration is monitored by intracellular recording from PNs postsynaptic to regenerated ORNs and by extracellularly recording oscillating local field potentials from the mushroom body calyx (Figure 1). After AN crushing, the size of the AL is reduced by 30% within 3 days (Stern et al., 2012). Anterograde labeling of the antennal afferents resolves the initial axotomy of the receptor cell axons. By scraping-off tips of olfactory sensilla on single flagellar annuli, only a few receptor cell axons can be dye-labeled (Wasser et al., 2017). This method reveals in finer detail that, similar to Wallerian degeneration in vertebrates (Waller, 1850), the axons distal to the crush degenerate. After 3 days post crushing, single fibers can be resolved which begin to regrow beyond the crush site in the antennal nerve. Reinnervation of the AL commences on 4 days post crush. After 1 week, large numbers of regenerating ORN axons start to grow around the borders of the glomerular neuropil. This leads to an enlargement of the AL, which returns to normal size within 2 weeks (Stern et al., 2012).

## Expression of Cell Surface Molecules During Development and Regeneration

Details of the regeneration process in the olfactory neuropils (Stern et al., 2012) were resolved by using monoclonal antibodies against cell surface proteins that were initially discovered as axonal guidance molecules in the ventral nerve cord of the locust embryo. Here, we will briefly describe their distribution pattern during brain development before monitoring their expression during regeneration. Fasciclin I (Fas I) is a neural cell adhesion molecule (Bastiani et al., 1987). After completing half of embryogenesis, antennal pioneer neurons and clusters of mushroom body neuroblasts are labeled by the Fas I antibody (Eickhoff and Bicker, 2012). During further development, the Fas I-immunoreactivity increases in the antennal nerve, AL neuropil, and a growing number of Kenyon cells. Fas I expression persists in ALs and mushroom bodies of larval and adult brains. An ortholog of the guidance molecule semaphorin 1a (Sema 1a), originally discovered by its function in pioneer axon navigation (Kolodkin et al., 1992; Isbister et al., 1999), contributes to the sorting of ORNs to different glomeruli in the developing AL of *Drosophila melanogaster* (Komiyama et al., 2007). During embryonic development of the locust, this transmembrane glycoprotein is also transiently expressed in ORNs and all olfactory neuropils (Eickhoff and Bicker, 2012). Another useful developmental marker is lachesin, a GPI-linked protein of the immunoglobulin family, that is expressed on the surface of developing locust neurons (Karlstrom et al., 1993; Boyan and Ehrhardt, 2020), including ingrowing larval ORN axons (Eickhoff and Bicker, 2012).

After experimental injury of the antennal nerve, Fas I expression fades in the AL neuropil until 4 days post crush, but remains unaltered in the mushroom bodies (Stern et al., 2012). Subsequently, Fas I-positive ORN axons begin to reinnervate the glomeruli of the AL. Twenty-one days post operation, the immunofluorescence intensity of glomerular staining becomes almost identical on treated and control sides. Regenerating fibers transiently express lachesin, indicative of a recapitulation of developmental programs. By day 21 post crush, lachesin labeling in the antennal nerve and lobe has completely vanished. Sema 1a expression shows that the neuropil of the AL retains its basic glomerular structure during deafferentation and regeneration. The immunofluorescence of this marker is rather uniformly distributed over the glomeruli of the AL and persists after the operation (Stern et al., 2012). Thus, the neuroanatomical branching pattern of PNs and local interneurons of the AL appears rather undamaged. Due to the conserved glomerular architecture during degeneration, the AL size reduction seems to be mainly caused by disintegration of the distal segments of ORNs.

## Structural Brain Plasticity After Sensory Deprivation

**FIGURE 2:**
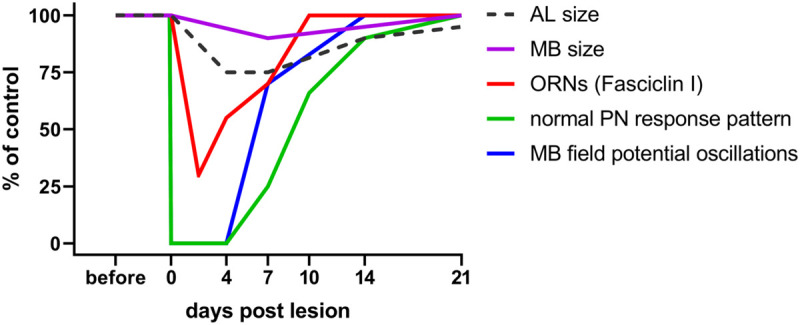
Time course of plastic changes in the adult locust olfactory system after lesion. Values are expressed as percentage of the unlesioned situation (before). On the neuroanatomical level, neuropil volumes and the cell surface protein fasciclin I indicative for ORN terminals in the AL decrease within the first 4 days post lesion, and increase back to normal within 21 days. On the electrophysiological level, responses are completely absent directly after the lesion, begin to return after 4 days, and return back to normal within 21 days as well. For technical reasons in a session of intracellular recordings from AL projection neurons, the chance to hit one of the very few (out of 830) cells with regenerated input is rather low. The mushroom body, however, integrates over all PNs. Thus, the chance to detect a regenerated input is much higher, explaining earlier re-appearance of mushroom body signals. Data from Eickhoff et al. (2012); Wasser et al. (2017), and Wasser and Stern (2017).

Does deafferentation of antennal input only affect the volume of ALs or also the target area of the PNs in the mushroom body? The consequences of nerve crush and complete antennal ablation on AL and mushroom body size were analyzed on days 7, 14, and 21 post operation (Eickhoff et al., 2012). Whereas after crush, ALs shrink within the first week, and grow back to normal size after that (Figure 2), they continue shrinking after antennal ablation, losing as much as 60% of their initial volume within 21 days. Measuring the calyx diameter revealed shrinkage to 86% of the original size within 21 days after antennal ablation. After nerve crush, a slight but significant decline in calyx diameter (95% of the control) is detectable after 7 days, but no significant size differences to controls remain after 14 and 21 days (Eickhoff et al., 2012; Figure 2). This result suggests the occurrence of regenerative processes within the olfactory pathway at later stages. To summarize, sensory deprivation of the primary olfactory center causes shrinkage of the mushroom body calyx, a transsynaptically induced structural change in a second order information processing neuropil.

## Precision and Misrouting of Regenerating Fibers

**FIGURE 3:**
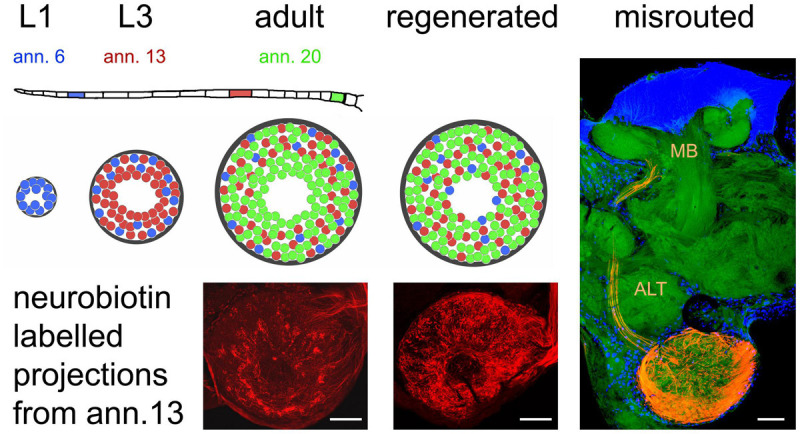
Organization of olfactory receptor neuron projections into the antennal lobe during development and regeneration. The flagellum of the locust antenna consists of 21 rings or annuli (ann.), which increase in number and size during postembryonic development through five larval stages (L1, L3 as examples) till adulthood. Receptor neurons terminate in a distributed fashion in the available glomeruli of the antennal lobe, which also gains size during development. This leads to a weak topographic pattern where older neurons born in younger stages (L1, blue) tend to terminate in the periphery, younger neurons born later (L3, red) terminate peripherally and medially, and terminals of younger neurons born just before the final molt (adult, green) are evenly distributed all over the antennal lobe. After regeneration, this pattern is absent. The photomicrographs of the neurobiotin-labeled sections correspond to the red glomeruli in the schematic. Only a small fraction of receptor neurons (∼15 axons when labeling the complete antennal nerve) fail to terminate in the antennal lobe and are misrouted through the antennal lobe tract (ALT) to the mushroom body (MB). Photomicrographs from Wasser et al. (2017). Scale bars 100 μm.

The olfactory pathway of the locust is capable of fast regeneration on a structural level, both at the first order neuropil of the AL and the second order neuropil of mushroom body. How precise is the process of AL re-innervation? Anterograde labeling of a small number of afferents on a single antennal annulus reveals a weak topographic match between ORN origin on the antenna and the position of innervated glomeruli (Wasser et al., 2017; Figure 3). Fibers originating distally on the antenna tend to innervate more peripherally located glomeruli, whereas projections from proximal parts of the antenna are more or less evenly distributed over the AL (Wasser et al., 2017). This pattern is most likely due to the sequence of postembryonic antennal development and growth. The first instar locust hatches with 11 antennal annuli, whereas the adult bears 21 of them. Not only the number of annuli but also their size increases between molts and the number of olfactory sensilla increases alongside (Ochieng et al., 1998). Between larval molts, proximal annuli divide, annuli in the middle of the antenna grow in size, and most distal annuli do not change much (Chapman and Greenwood, 1986; Chapman, 2002). Thus, many new ORNs are generated on proximal, some on medial, and hardly any on the most distal annuli. At the same time, number and size of AL glomeruli increase, with many new glomeruli arising in the center of the AL, and fewer intercalating between existing microglomeruli (Anton et al., 2002). Therefore, most of the oldest glomeruli innervated mostly by ORNs originating distally on the antenna end up in the periphery of the AL, whereas younger glomeruli, innervated by ORNs from the proximal part are rather evenly scattered over the AL neuropil (Figure 3). This antennotopic pattern is, however, less clearly defined than e.g., the topographic representation of mechanosensory afferents in the locust thoracic ganglion (Newland, 1991), and whether it has any functional significance is not clear. Anton and Hansson (1996) reported no correlation between PN response characteristics and the distance of the arborizations from the central fiber core. In preparations with lesioned nerves, the antennotopic arborization patterns of ORNs did not reappear (Wasser et al., 2017; Figure 3). Nevertheless, the vast majority of ORNs terminated in the AL, which is quite unusual. Generally, pathfinding errors are very common for regenerating axons (Chiba and Murphey, 1991; Lakes and Kalmring, 1991; Lakes-Harlan and Pfahlert, 1995; Stern et al., 1997; Stern and Bicker, 2008; Krüger and Lakes-Harlan, 2010). For instance, in the regenerating auditory pathway of locusts, even as many as 70% of the regenerated fibers are misrouted (Jacobs and Lakes-Harlan, 2000). Thus, the precise termination of most regenerating ORN axons in the AL is an exception. A few axons (up to 15) fail to terminate in the AL, however, they leave the AL via the antennal lobe tract (ALT), arborizing in the mushroom body calyx and the lateral horn, areas that are normally innervated by the PN axons running in this tract (Figure 3). Rössler et al. (1999) have described such a misrouting of antennal afferents during pupal development of the hawk moth *Manduca* with experimentally damaged sorting zone glia. However, fibers had also grown incorrectly into other brain regions under their experimental circumstances, whereas misrouted regenerating locust ORN axon were confined to components of the olfactory pathway (Stern et al., 2012). One explanation could be that the ALT is the only exit from the AL, which is, like the ALT itself, enveloped by glial processes (Hähnlein et al., 1996). Possibly, the rather unique multiglomerular architecture of the acridid *Locusta* AL as compared to other, even closely related insects (Ignell et al., 2001), enhanced the chance for successful functional regeneration.

## Physiological Proof of Regenerating Synapses

Successful regeneration requires not only the re-innervation of the correct target but also recovery of physiological function. The olfactory system of the locust is quite amenable to studies of functional recovery by electrophysiological recording of field potentials and intracellular recordings at the level of antennal receptor cells, AL neurons, and mushroom body neurons (Laurent and Naraghi, 1994; Anton and Hansson, 1996). When recording from PNs in the ALs of axotomized locusts, the first few odor stimulus evoked postsynaptic responses from regenerated ORNs occurred from days 4 to 7 post crush on, matching well to the observations of regeneration on the neuroanatomical level (Figure 2). The proportions of response categories (excitatory vs. inhibitory) changed during regeneration, but were back to normal within 21 days (Wasser and Stern, 2017; Figure 2). Odor-evoked oscillating extracellular local field potentials (LFP) were recorded in the mushroom body (Figure 1). These responses, absent after antennal nerve crush, reappeared, in a few animals as soon as 4 days post crush. Odor-induced oscillation patterns were restored within 7 days post crush (Figure 2). Both intra- and extracellular recordings indicate the capability of the locust olfactory system to re-establish synaptic contacts in the AL after antennal nerve lesion. Thus, electrophysiological analysis at the systems level shows plastic recovery of synaptic circuitry.

Behavioral studies of odor discrimination after regeneration are still lacking. Whether the animals could reacquire the lost sense of smell, and discriminate odors again, could only be tested by behavioral choice experiments as recently developed by Simoes et al. (2011).

## The Influence of Age on Regeneration

In the vertebrate CNS, age is an important factor that determines the extent and speed of regeneration after injury. Of course, we wondered also about the precision and age-dependence of the regeneration process in the locust olfactory system. Both ORN distal segment degeneration in the AL and regeneration of ORN axons measured by FAS I-immunofluorescence were significantly faster in 5th instar nymphs than in adults (Wasser et al., 2017). Accordingly, re-establishment of ORN-PN synapses was significantly quicker in nymphs than in adults. Finally, the restoration of the AL network, judged by correct ratios of inhibitory to excitatory inputs to the PN population, was also faster in 5^th^ instars than in adults (Wasser and Stern, 2017). Faster or more effective regeneration has also been demonstrated in other sensory pathways in insect preparations, e.g., the auditory nerve of locusts (Lakes et al., 1990) and bush crickets (Krüger et al., 2011a,b), as well as the peripheral nerve of cockroach (Guthrie, 1962). The shorter distance in the slightly smaller 5th instar can only partly explain faster regeneration. An explanation could be that cell intrinsic factors limit the regeneration capabilities of older neurons as observed in the mammalian spinal cord (Blackmore and Letourneau, 2006). However, we found no evidence for limited regeneration of the older ORNs originating on the peripheral antennal annuli as compared to younger, proximal ORNs. A different explanation could be that the subadult antennal system is still in a state of growth and rearrangement that may be more permissive for ORN regeneration and synapse formation than the adult AL. In the silk moth and in *Drosophila melanogaster*, molting hormones (20-HE) in the hemolymph have been shown to enhance neurite outgrowth both *in vitro* (Kraft et al., 1998) and *in vivo* (Zwart et al., 2013).

## Mechanistic Approaches to Regeneration

We have introduced the olfactory pathway of the locust *Locusta migratoria* as a model system for neural regeneration demonstrating that axonal regeneration in this system is possible and beyond that, fast and precise (Stern et al., 2012). Differences between experimental groups based on age can be quantified on the neuroanatomical and physiological level (Wasser et al., 2017; Wasser and Stern, 2017). Is it possible to manipulate the regeneration process in a way that function of the neural circuitry can be more efficiently restored? Currently it is too early to provide a conclusive answer, but some results from other labs and our own are outlined. A convenient way to manipulate regeneration in the locust are gain-of-function experiments in which drugs or natural ligands are applied during regeneration. After injection into the animals or bath application to cell cultures, neuronal responses can be quantified. For example, addition of 9-cis retinoic acid to dissociated cell cultures of embryonic locusts causes the extension of more neurites per neuron (Sukiban et al., 2014). Application of recombinant human erythropoetin facilitates the regeneration of neurites in dissociated locust brain neurons and, using a behavioral assay with the grasshopper *Chorthippus biguttulus*, accelerates reestablishment of sound source localization after unilateral crush injury of the peripheral tympanic nerve (Ostrowski et al., 2011).

During embryonic development, the nitric oxide/cGMP system facilitates axon extension in antennal pioneer neurons (Seidel and Bicker, 2000) and the migration of enteric neurons on the midgut (Haase and Bicker, 2003; Stern et al., 2007; Stern and Bicker, 2010). In both cases, nitric oxide induces specific neurons of the PNS to raise the intracellular cGMP level, which in turn causes a reorganization of the cytoskeleton leading to increased cell motility. Based on these findings, axonal regeneration in the CNS of locust embryos was also addressed in a culture system for an embryo preparation in which the CNS is exposed to chemical compounds targeting the NO/cGMP system and the connectives between abdominal ganglia were crushed (Stern and Bicker, 2008). To analyze the axonal response to injury, changes in the anatomy of serotonergic multisegmental interneurons by immunocytochemistry after crushing ventral nerve cord connectives were followed. After initial retraction, serotonergic axons grow back into the adjacent ganglion within a few days. Regeneration rate is improved by NO donors and reduced by inhibitors of the NO-cGMP pathway. Since the regenerating serotonergic neurons express NO-induced cGMP-immunoreactivity, they are presumably the targets of NO signaling (Stern and Bicker, 2008). Most likely, axons of nitrergic neurons damaged by the crush procedure are a source of internal NO. After crushing of connectives, the accumulation of citrulline, a by-product of NO synthesis, is visualized in nitrergic neurons by immunocytochemistry (Stern et al., 2010). In the presence of NO-scavengers, we find reduced regeneration of injured axons, suggesting that NO from internal sources enhances the regeneration process (Stern and Bicker, 2008). In the nervous system of the locust, the AL contains the highest amount of Ca^2+^/calmodulin-dependent NO synthase activity (Müller and Bicker, 1994; Elphick et al., 1995). The source of NO is a group of nitrergic interneurons which innervate all glomeruli. It might be rewarding to find out whether the functional regeneration of the insect olfactory pathway could also be improved by NO donors or other external stimulators of the NO/cGMP cascade.

## Outlook

Does our research on an invertebrate animal have implications for neuroregenerative therapies in human patients? A particular challenge provides spinal cord injury, in which the non-permissive tissue environment and neuron intrinsic factors restrict the regeneration of injured axons. In the mammalian nervous system, the limited regenerative response to spinal cord injury is usually considered to involve a different cyclic nucleotide pathway, the cAMP/protein kinase A signaling cascade (Hannila and Filbin, 2008). Along similar lines, an investigation of human model neurons in cell culture confirmed that neurite outgrowth is indeed enhanced by activating cAMP/Protein kinase A signal transduction (Tegenge et al., 2011). These results argue against excessive generalization of concepts derived from phylogenetically very distant animals. However, it is well known that even in the vertebrate CNS, growth responses of neurites depend on the species, specific part of the brain, and neuron type. In lower vertebrates, such as fish and amphibians, the regeneration capacity of the CNS is certainly higher than in mammals, which cannot regenerate injured axons (Ramon y Cajal, 1928; Rossignol et al., 2007).

For example, the olfactory system of the African clawed frog Xenopus provides ample opportunities for studying mechanisms of regeneration (Yoshino and Tochinai, 2006). Here, neurogenesis during development and a lifelong neuronal turnover in the olfactory epithelium are essential contributions to the regenerative capabilities of the olfactory circuitry. Transsection of the fully functional olfactory nerve of Xenopus larvae causes degeneration of ORN in the sensory epithelium which is followed by cell death in the postsynaptic olfactory bulb and reduction of its volume (Hawkins et al., 2017). The neural injury induces an increase in epithelial stem cell proliferation and newly formed ORN start to reinnervate the olfactory bulb within 1 week. Olfactory network reconstruction, as assayed by cellular calcium imaging in the olfactory bulb, appears to recover within 7 weeks (Hawkins et al., 2017).

Another striking example is the regeneration of the amphibian optic nerve after injury, which led to intriguing experimental studies culminating in the chemoaffinity hypothesis of Sperry (1963). This hypothesis proposed that developing neurons systematically acquire chemical identification tags, by which they selectively recognize their correct targets. Optic nerve injury in the goldfish causes induction of the neuronal NO synthase enzyme (Koriyama et al., 2009) and the resulting NO release promotes neurite outgrowth similar to locust ventral nerve cord regeneration. Again, axonal regeneration of the fish retinal ganglion cell axons is significantly enhanced by pharmacological activators of NO/cGMP signaling (Koriyama et al., 2009). Downstream targets of NO/cGMP signaling during neuroregeneration have to be identified, but there are likely candidates. The second messenger cyclic GMP can activate protein kinase G (PKG) which is also implicated in the regeneration of retinal ganglion cell axons (Koriyama et al., 2009). During mouse development, NO and cGMP positively regulate neurite length and migration of immature neurons derived from the medial ganglionic eminence (Mandal et al., 2013). An increase in cellular motility is mediated by PKG which, in turn, downregulates the RhoA signaling pathway and eventually is thought to affect the cytoskeleton via myosin light chain phosphatase (Mandal et al., 2013). The RhoA pathway is one of the key elements responsible for the restricted regeneration capacity of the mammalian CNS (Mueller et al., 2005).

In the mammalian CNS, extracellular growth inhibiting factors bind to several specific receptors on the axonal growth cone and converge on the small GTPase RhoA signaling pathway (Tönges et al., 2011). In the active state, RhoA interacts with downstream effector proteins such as Rho-associated coiled coil forming protein serine/threonine kinase (ROCK), thereby leading to the phosphorylation of various target proteins which regulate mainly actin cytoskeletal dynamics (Mueller et al., 2005). Experimental studies in rodents show that inhibition of RhoA-ROCK overcomes the inhibitory effects of myelin and chondroitin sulfate proteoglycans, resulting in increased axonal regeneration in cell culture and improved fiber sprouting across the lesion site (Dergham et al., 2002; Fournier et al., 2003; Lingor et al., 2007). High concentrations of the analgesic, Ibuprofen decrease RhoA activation and promote neurite growth in cell cultures of human model neurons (Roloff et al., 2015). Inhibition of the downstream effector ROCK by the drugs Y-27632 and Fasudil enhances the neurite growth capacity (Roloff et al., 2015). In line with these encouraging results, the inhibitory effects of the pesticide rotenone on axon extension of locust pioneer neurons can be rescued by co-application of the ROCK inhibitor Y-27632 (Bergmann et al., 2019). Since neurite extension is one of the essential components of regeneration, experimental results from mammals and the locust suggest that molecules of the RhoA/ROCK signaling cascade are useful targets for identification of pharmaceutical compounds with therapeutic benefit for the treatment of spinal cord injury.

## Author Contributions

GB and MS wrote the manuscript. Both authors agreed to be accountable for the content of the work.

## Conflict of Interest

The authors declare that the research was conducted in the absence of any commercial or financial relationships that could be construed as a potential conflict of interest.
